# Supplementary material for: Experience of Pharmacists with Anti-Cancer Medicine Shortages in Pakistan: Results of a Qualitative Study
Source: Int J Environ Res Public Health. 2022 Dec 6;19(23):16373. doi: 10.3390/ijerph192316373 (PMC9740989; doi:10.3390/ijerph192316373)
Supplement: Supplementary file 1 [file ijerph-19-16373-s001.zip › ijerph-2021474-supplementary.pdf]

Supplementary Materials File S1: Questionnaire for "Experience of pharmacists with anti-cancer medicine shortages in Pakistan: results of a qualitative study"

Supplementary file

INTERVIEW GUIDE FOR ONCOLOGY PHARMACIST

<table>
<tr><th>Discussion Topics</th><th>Details</th></tr>
<tr><td>1. General information</td><td>a. Gender:__________________
b. Age:__________________
c. Years of anticancer medicine related experience: _________
d. Type of hospital: _____________________
e. Designation: _________________________
f. Email ID: ____________________________</td></tr>
<tr><td>2. What are your general thoughts about anticancer medicines shortages?</td><td>a. Do you experience anticancer medicine shortages in your practice?
b. How often do anticancer medicines shortages occur in your practice?
c. What ethical dilemma you have to face while dealing with chemotherapy shortages?
d. List specific names of anticancer medicines in shortage (generic name, brand name, strength, and dosage form) during the last six months in your hospital.

<table><tr><th>SR.NO</th><th>BRAND</th><th>GENERIC</th><th>STRENGTH</th><th>DOSAGE</th></tr><tr><td>1</td><td></td><td></td><td></td><td></td></tr><tr><td>2</td><td></td><td></td><td></td><td></td></tr><tr><td>3</td><td></td><td></td><td></td><td></td></tr><tr><td>4</td><td></td><td></td><td></td><td></td></tr><tr><td>5</td><td></td><td></td><td></td><td></td></tr></table></td></tr>
<tr><td>3. What are the reasons for cancer medicines shortages?</td><td>a. What are the reasons for cancer medicines shortages from the perspectives of medicine manufacturers?
b. What are the reasons for cancer medicines shortages from the perspectives of pharmaceutical distributors or wholesalers?
c. What are the reasons for cancer medicines shortages from the perspectives of healthcare institutes?
d. Are there any other reasons causing cancer medicines shortages?
e. Among those causes mentioned above, what are the three most important reasons in your opinion?</td></tr>
<tr><td>4. What are the effects of cancer medicines shortages on patients (care outcome) and pharmacists?</td><td>Patients:
1) Financial consequences
2) Clinical consequences
Pharmacists:</td></tr>
<tr><td>5. What action(s) did you take to manage chemotherapy shortages?</td><td></td></tr>
</table>

6. How can we manage or solve the medicines shortage problem in your opinion?	a. What could the governmental authorities do to manage cancer medicines shortages?<br>b. What could the drug manufacturers do to deal with cancer medicine shortages?<br>c. What could the distributors or wholesalers do to deal with cancer medicines shortages?<br>d. What could the healthcare institutions do to deal with cancer medicines shortages?<br>e. Are there any other solutions to manage this problem?<br>f. Among those solutions, what are the three most important solutions in your opinion?
